# Application of 3D hologram technology combined with reciprocal style to learn some fundamental boxing skills

**DOI:** 10.1371/journal.pone.0286054

**Published:** 2023-05-23

**Authors:** Ahmed Hassan Rakha

**Affiliations:** Department of Curriculum and Teaching Methods of Physical Education, Faculty of Physical Education for (Men–Girls), Port-Said University, Port-Said, Egypt; Politecnico di Torino, ITALY

## Abstract

The use of 3D Hologram Technology (3DHT) in teaching and learning has many advantages, such as attracting students’ attention, reducing cognitive load and self-effort, and improving spatial awareness. In addition, a number of studies have confirmed that the reciprocal teaching style is effective in teaching motor skills. Thus, the current study aimed to investigate the effectiveness of reciprocal style when combined with 3DHT in learning some fundamental boxing skills. A quasi-experimental design was used by creating two experimental and control groups. For the experimental group, the reciprocal style is combined with 3DHT to teach some fundamental boxing skills. In contrast, the control group is taught a program based on a teacher command style. Pretest-posttest designs were made for the two groups. The sample consisted of 40 boxing beginners aged 12 to 14 who were enrolled in the 2022/2023 training season at Port Fouad Sports Club in Port Said, Egypt. The participants were randomly divided into two experimental and control groups. There were homogenized based on age, height, weight, IQ, physical fitness, and skill level. The results showed the experimental group achieved a higher skill level due to the combination of 3DHT and a reciprocal style in compared to the control group, which relied solely on the teacher’s command style,. Due to this, it is imperative to utilize hologram technology as a pedagogical resource to enhance the learning process and combine it with teaching strategies that support active learning.

## Introduction

Technology has enabled students and teachers to have interactive and engaging learning experiences, and providing teachers with more advanced teaching strategies boosts the educational environment. Animation is one of the most effective tools for creating engaging learning experiences for students, as it can bring topics to life and make them easier to understand. Additionally, animation can help keep students motivated and interested in the learning process, which can help them retain information better. Based on Korakakis et al. [1], animation is a valuable tool for communicating services or spreading ideas as it helps learners quickly understand and learn. It facilitates the learning process, simplifies information, and increases learner retention by explaining relationships between objects and ideas. Barnea [2] suggested that objects are better understood when represented, rotated, and reflected in three dimensions (3D). Milik et al. [3] suggested that spatial ability is an important factor in the use of multimedia in education because learners need to absorb spatial information from sensory memory, maintain internal schemas (knowledge structures) in working memory, and make spatial transitions to consolidate information into long-term memory. Barnea [2] categorized visualization skills into three categories: spatial visualization, orientation, and relations. Spatial visualization involves understanding 3D objects through their 2D representations, spatial orientation involves envisioning what a representation would look like from a different perspective, and spatial relations involve manipulating objects mentally.

Korakakis et al. [4] demonstrated that multimedia applications with 3D interactive animation increase students’ interest and make learning objects more appealing, enabling them to control their learning time. Hoyek et al. [5] found that 3D digital animations effectively teach human anatomy, particularly in recalling anatomical knowledge that requires spatial ability. Wu and Chiang [6] explained that students better understand visual concepts when 3D animation is used. CAKİROGLU and YİLMAZ [7] found that video clips and 3D animations reduced misconceptions about computer systems and enhanced understanding of concepts by allowing students to render, pause, slow down, restart, and extend features. These studies relied on the use of computers to display 3D movies, whereas in the current study, 3D hologram technology (3DHT) will be used, which is one of the technologies that display 3D images and videos with many features, including the ability to present information more realistically. The clarity and attractiveness of the method make it an appealing alternative to traditional ways of clarifying information. Learning with 3DHT can increase students’ attention, reduce cognitive load, and improve spatial awareness [8–11].

Boxing is a combat sport that depends on physical factors, technical skills, tactical insight, and mental strength. For beginners, the mental aspect is the most important to increase physical, technical, and tactical effectiveness. Cognitive awareness is key to developing psychomotor skills, spatial awareness, and motivation to learn. A correct understanding of boxing skills contributes to improving educational and training processes and reaching optimal motor skills [12]. Boxers who master the fundamental skills of boxing are much better at linking attack, defense, and counterattack. Boxing studies such as [13–16] have revealed that beginners tend to make many mistakes, which can be treated and corrected using various teaching scaffolds. It was found that watching educational movies about the skill, especially 3D ones, was the most helpful for increasing beginners’ awareness of the correct sequence of skill performance. The current study uses hologram technology to provide 3D movies for illustrating fundamental boxing skills, enhancing students’ spatial awareness. Although the hologram fan has been used in many educational settings, it is still being investigated for teaching motor skills in sports. This especially benefits boxing beginners because they can observe a professional boxer under real-life conditions and enhance their motor awareness. In light of 3DHT’s advantages and the need for cognitive awareness in teaching boxing to develop physical, technical, and tactical skills, the current study attempts to bridge the gap between the coach’s needs in teaching beginner boxers fundamental skills, increasing motivation for learning, mastering those skills, and developing cognitive, motor, and spatial skills. Since most boxing exercises are in pairs, with reciprocal attacks and defenses between them, the 3DHT has been combined with a reciprocal teaching style. Within the Spectrum of Teaching Styles, reciprocal teaching is an essential direct style aimed at maximizing social learning through discussions between the learner and their partner to exchange feedback and achieve optimal performance [17]. Several studies have confirmed the effectiveness of the reciprocal teaching style in teaching motor skills [18–20]. Thus, the current study aims to investigate the effectiveness of the reciprocal teaching style when combined with 3DHT in learning some fundamental boxing skills.

Therefore, the current study aims to answer the question: what is the effectiveness of using the reciprocal teaching style when combined with 3DHT on students’ performance level of basic boxing skills?

From this main question, the following sub-questions emerge: is there a statistically significant difference between the pre- and post-measurements of the experimental group regarding fundamental boxing skills; what is the significance of the differences between the experimental and control groups on the post-measurement of fundamental boxing skills; and what is the effect size of the reciprocal style combined with 3DHT?

## Theoretical framework

### The constructivist cognitive development theory

Constructivist cognitive development is the dominant paradigm of educational development, based on Piaget’s cognitive development and Vygotsky’s sociocultural theory. This theory emphasizes that students construct their own knowledge, not that teachers transmit it to them. Through constructivism, teachers become guides and counselors for students, presenting problems and requesting them to identify and analyze details, data, and relationships, thus constructing a cognitive structure. Teachers organize learning environments, provide tools, manage and evaluate learning, and provide back-up information to their students [21].

According to Merriam et al. [22] in constructivist theory, learning is generative, creating meaning from experience. According to constructivism, each learner constructs knowledge based on their own experience and interactions with others. As a result, a learner gains knowledge and becomes more dynamic. A constructivist-learning approach asserts that knowledge is our own construction, and the learner is the center of the educational process. The learner discusses the problem, gathers information, discusses the proposed solutions with his colleagues, and then studies the possibility of applying these solutions scientifically. Knowledge is the result of the cognitive construction of reality through one’s activities.

Oxford [23] outlined five stages of constructive learning:

1. Initiation of new knowledge.
2. The process of acquiring new knowledge begins with learning as a whole, then paying attention to details.
3. Developing knowledge by building tentative hypotheses, discussing them with colleagues in order to obtain answers, and then developing and modifying that knowledge based on collaborative responses.
4. Putting knowledge into practice and gaining experience.
5. Considering the development of knowledge.

According to Oxford [23] constructive learning aims to create freedom, realism, positive attitudes, and perceptions of education as capital, essential for students to learn effectively. By using modern technological resources, teachers support a knowledge-constructing environment that takes into account the learner’s tendencies, needs, and thinking processes.

### Sociocultural constructivist theory

Lev Vygotsky developed this theory, which emphasizes that knowledge exists in a social context [24]. Eggen and Kauchak [25] clarified that directed social situations are essential to learning. There are two types of social interdependence between individuals, according to Johnson and Johnson [26]: (1) positive interdependence, which is characterized by individuals pursuing common goals, and (2) negative interdependence, in which individuals block each other’s goals. Therefore, the teacher promotes social interdependence among her students so they can achieve the desired knowledge structure on their own. Haenen et al. [27] proposed that children could learn when they engage in meaningful activities with intelligent individuals. Vygotsky’s theory states that the ability of a learner develops over two levels: (1) the social level, which refers to the social educational environment in which learners can learn, (2) the learner’s psychological level, which refers to the psychological and mental processes that occur in the learner’s mind when interacting with the social educational environment.

**Fig 1:**
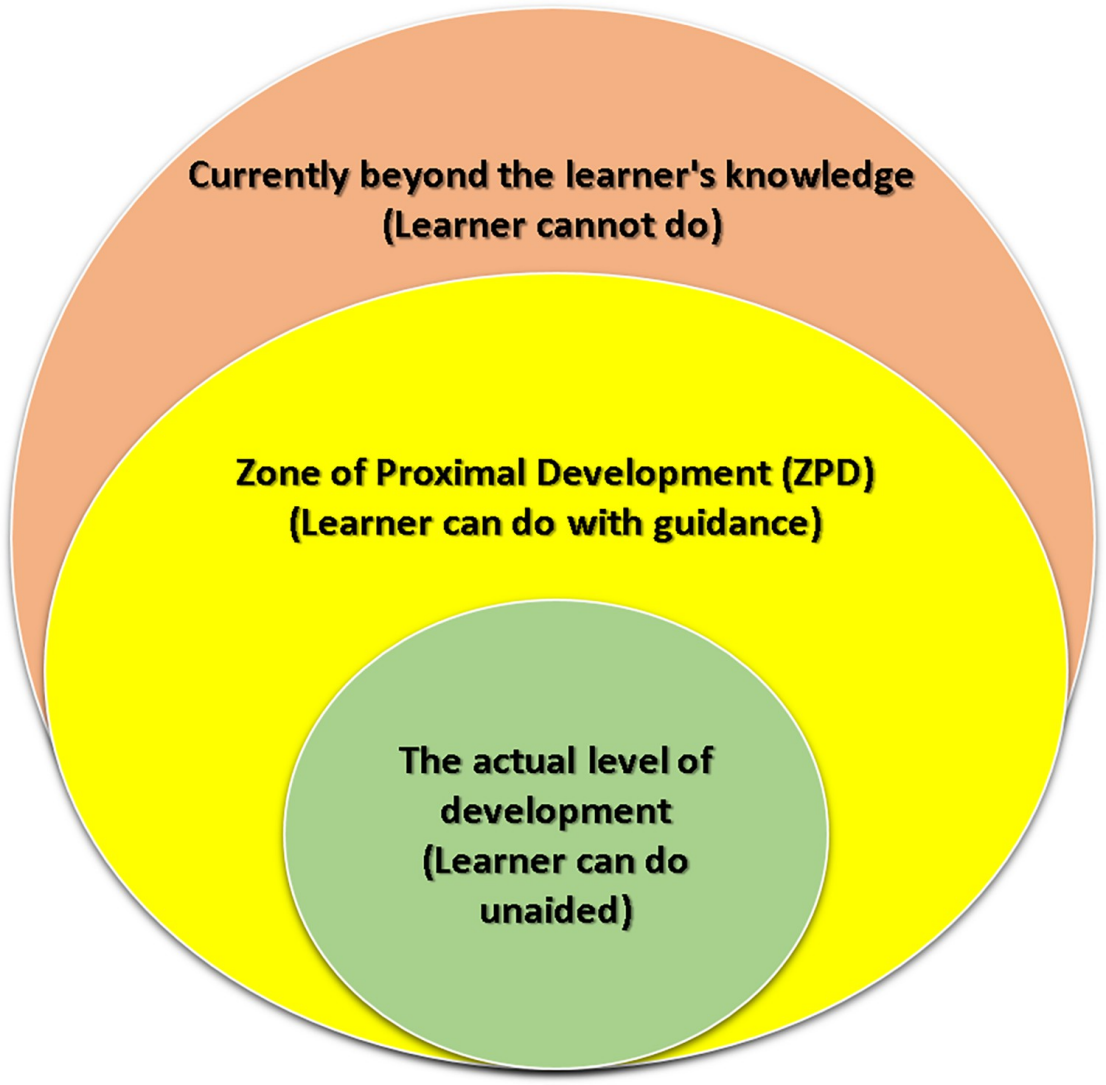


According to Vygotsky and Cole [28], a zone of proximal development (ZPD) as shown in (Fig 1) is a level of development between actual and potential development. The actual level of development can be observed through the learner’s completion of tasks and solving various problems independently. This is called the internal mental capacity of the learner. While the potential level of development is seen in the learner’s ability to complete tasks and solve problems by guiding a teacher or when cooperating with more competent peers. This is called the learner’s external mental capacity. ZPD is defined as functions or abilities that have not fully developed and are still in the process of maturing. In order to explain the concept of ZPD, the term scaffolding is used. Since the ZPD is viewed as a scaffold, it serves as a starting point. Vygotsky and Cole [28] define scaffolding as the metacognitive, strategic, or procedural support that allows learners to participate in activities and build their skills.

Berk and Winsler [29] suggested implementing steps to ensure the success of educational scaffolds:

1. Students’ ZPD is explored through quizzes or discussions to determine what they already know about the learning topic.
2. Various abilities of students are grouped into teams to increase their learning potential. Make sure that every member of the group participates actively.
3. Vygotsky’s scaffolding can be counterproductive when the teacher provides students with too much assistance, making them passive rather than engaged.
4. Students are encouraged to think aloud to determine their current abilities, thereby determine their ZPD and to ensure active learning.

### Cognitive load theory (CLT)

Working memory ability is a crucial determinant of academic achievement, according to the CLT. As working memory has a finite capacity, it often becomes overwhelmed while processing complicated instructional content. Working memory has to be able to withstand the demands of learning in order to learn well. Therefore, instructional methods should be carefully structured to exert the right amount of cognitive burden on working memory in order to assure learning success [30, 31].

Sweller [30] described two types of cognitive load: useful (intrinsic, germane) and unnecessary (extraneous). Intrinsic load is the mental effort required to learn new information, retain it in working memory, and process it. It plays a significant role in determining how effectively a student can complete a task. Germane cognitive load is the term used to describe the mental effort required to make sense of the content being studied and to create mental models, or frameworks, for comprehending and remembering it. While this type of cognitive load requires more working memory, it helps information to be retained in long-term memory. Extrinsic cognitive load occupies working memory, which includes tasks unrelated to comprehension, schema creation, or process automation. The problem can arise from too much complexity in educational material, a large amount of information, or an unfamiliar educational environment. As a result, learners may feel frustrated and find it difficult to complete their tasks [30–32]. Increasing external and internal loads may lead to an overloaded working memory [31].

### The spectrum of teaching styles in physical education theory

Muska Mosston developed the teaching styles spectrum in physical education in 1966 to ensure all students understood the fundamental concepts, abilities, and ideas of physical education. Spectrum is based on the premise that teaching behavior can be explained as a chain of decisions. There is always a previous decision behind every deliberate act of teaching. There are three types of decisions in each style: pre-impact, impact, and post-impact. A pre-impact set of decisions includes decisions regarding the lesson’s objectives, the teaching method, the subject matter, the classroom environment, the location of the lesson, organizational arrangements, grading policies, and time (e.g., start and end times). The impact decision set includes options for putting pre-impact decisions into practice and, if necessary, modifying them. Post-impact decision categories include gathering data on learners’ performance, evaluating students’ performance against standards, giving feedback to students, and evaluating teaching approaches. There are many different teaching styles on the spectrum, from teacher-led instruction to more student-centered instruction. Physical education teachers are able to implement PE programs in dynamic settings by utilizing a number of teaching styles [33]. The spectrum includes eleven distinct teaching styles, including five teacher-centered styles (command, practice, reciprocal, self-check, and inclusion), six student-centered (guided discovery, convergent discovery, divergent discovery, learner designed Individual Program, learner initiated, and self-teaching) [17]. In addition to the eleven landmark styles, there is a wide variety of styles called canopies. It is created by changing the anatomy of the style or by combining two landmark styles. Since canopies are positioned between the two landmark styles, they share the same decision configuration [34].

### Reciprocal style

The reciprocal style is one of the direct styles in spectrum theory. In this style, learners are organized in pairs, each with a specific role to play. As the doer, one learner performs the task. As observer, learner provides constant and immediate feedback to the doer using a criteria sheet designed by the teacher. At the end of the practice, the observer and the doer switch roles [17]. In Mosston and Ashworth [17] view, this teaching style can lead to the realization of the following strengths:

- By giving feedback to a peer, learners are able to do better because the observer provides feedback more frequently, which results in a higher number of correct responses.
- As learners receive and give feedback to a peer, their socialization skills expand.
- Observer analyse movements by monitoring the doer’s performance, comparing it against criteria, and drawing conclusions about the accuracy of the performance.

In the Reciprocal Style, the teacher’s (T) job is to decide on all subject matter, criteria, and logistical issues (pre-impact decisions), as well as to watch over students and provide them individual comments about their roles (post-impact statements). Throughout the learning process, learner (L) works in a partnership relationship. The doer (L_d_) makes the nine decisions of the Practice Style (impact decisions) on a task. As the observer (L_o_) provides immediate and on-going feedback to the doer about the task’s correctness (post-impact) using a criteria sheet designed by the teacher, the Lo is making the five decisions that are shifted in this style. Roles are exchanged between the partners [17, 20, 33].

### Instructional design models

Instructional design (ID) is a framework for integrating technology into education. It focuses on learners rather than teachers, making the program more applicable and meaningful to them. Koper [35] defined it as the teaching and learning process that takes place in a learning unit such as a course, lesson, or other designed learning event. It represents the learning and support activities undertaken by the teacher and the learner in the context of the learning unit. ID is the systematic process of planning events to facilitate learning. This process includes several interrelated steps such as analyzing learners, contexts, and objectives, developing strategies, designing evaluation tools, and producing educational materials. It was developed in the 1950s and 1960s by the military and business worlds and dominated education technology and education development in the 1970s. Over a hundred different ID models exist today, almost all of which are based on the general ADDIE model [36]. Some of the most well-known and commonly used models include ASSURE, DDD-E, Kemp, and Smith and Ragan.

### ADDIE model

**Fig 2:**
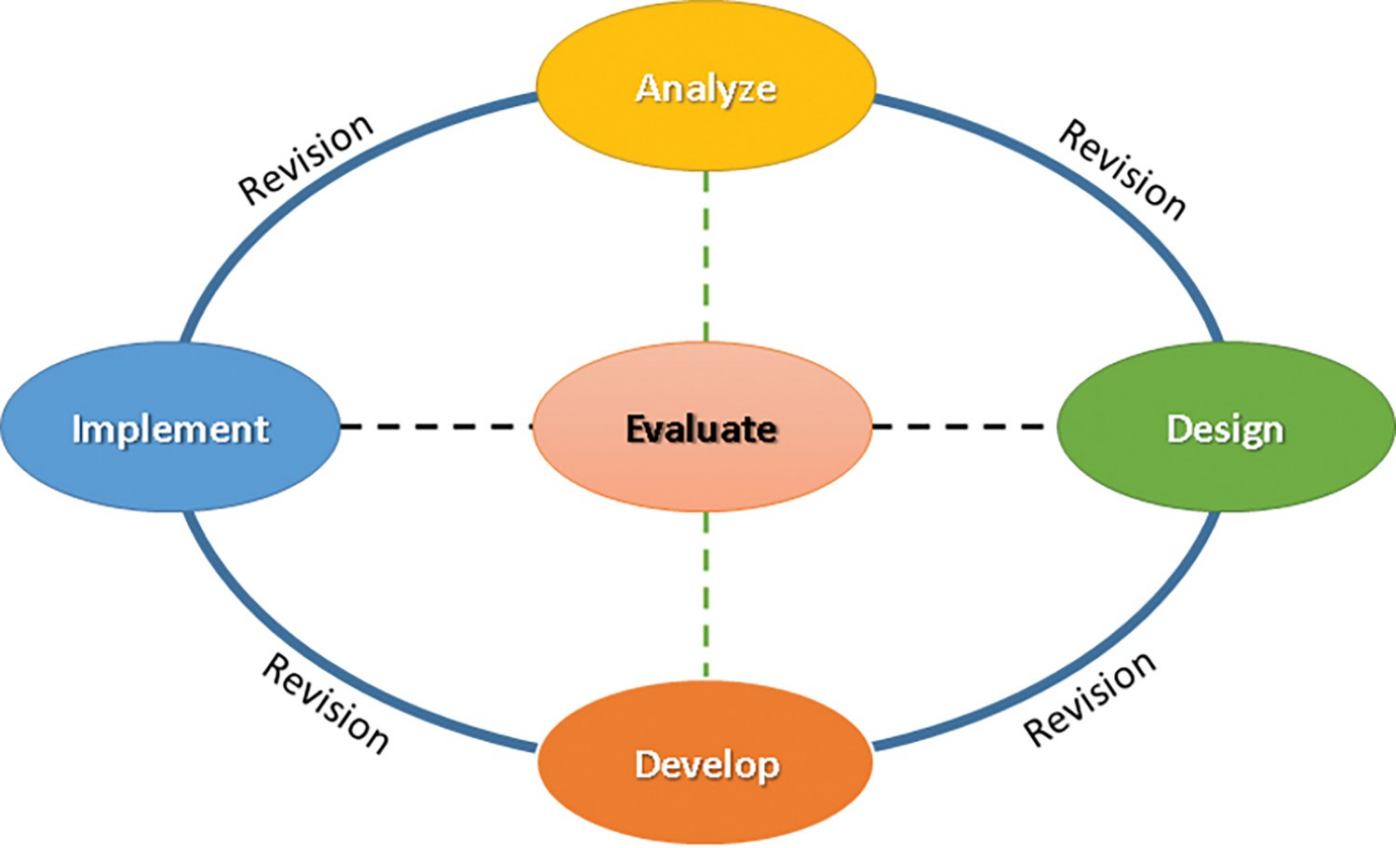


According to [37–39], the ADDIE model is one of the most popular instructional design models It’s a cyclical and iterative process that helps instructional designers to create effective training materials into manageable steps. As shown in (Fig 2), the acronym indicates five stages:

- *Analysis*: this includes students’ analysis, desired program objectives, learning objects, and tasks.
- *Design*: consists of learning outcomes, content, strategies, teaching methods, and activities for learning.
- *Development*: Interactive multimedia applications and learning-based technologies are used.
- Implementation: The process of delivering educational materials or educational program to students.
- *Evaluation*: As part of the model, evaluation is a critical element that is performed at each stage to ensure the educational process stays on track in all stages. This involves allowing students to enter various stages of the process as necessary based on the situation and ensuring that the teaching and learning process achieves the desired outcomes.

According to the Davis [40], the ADDIE model is useful for novices and inexperienced educational designers. In addition to [39, 41–44], the ADDIE model is comprehensive and can be used to develop educational programs with clear objectives, well-designed instructional strategies, and meaningful activities. Additionally, it can be used to evaluate and modify the program.

### 3D hologram technology (3DHT)

Gdoutos [45] explained the term Hologram is derived from the Greek words “holographos” and “gramma”, which mean “whole” and “message” respectively. Dennis Gabor introduced this term in 1947 to describe recording 3D images on film. Gabor’s invention revolutionized the field of imaging and has since been used in many different applications. A Hologram is a 3D representation of laser light wave interaction. According to Barkhaya and Halim [46], it involves spatial shows that are integrated into the real world. Furthermore, 3DHT provides a more immersive learning experience than traditional teaching methods, allowing students to interact actively with the learning objects. It helps students who have trouble paying attention in class to gain a deeper understanding of the learning objects. Hoon and Shaharuddin [47] explained that 3DHT creates 3D illusions for viewers related to audio, visual, and vertical content to simplify difficult concepts. This helps students monitor and imagine content while learning in addition to attracting their attention.

Raza and Sharma [48] Raza and Sharma (2012) provided an overview of the following 3DHT features:

- An eye sees 3DHT light the same way it sees light from an original scene.
- Since 3DHT are reconstructed waves, they are monochromatic rather than natural colour.
- When one looks through a 3DHT, they are given the impression of seeing in 3D, which is a genuine effect, not a psychological one.
- Depending on the laser’s coherence length, the 3DHT appears bright and detailed.

[10, 49] demonstrated several techniques of 3DHT, including the following:

- *The Reflection Hologram* is the most common type of hologram formed when the reference beam and object beam collide on opposite sides of the holographic surface. Images are recorded as a result of their interference, and a point source of white light illuminates the hologram from the proper angle. Reflection holograms require the simplest setup and are without laser light.
- *Hologram transmissions* are laser-transmission holograms created when the reference beam and object beam interact on the same side of the holographic surface. A spread-out laser beam is shone through the emulsion side, passing from behind the hologram device to the observer, creating a precise image.
- *Hybrid holograms* are a combination of transmission and reflection holograms, also known as multichannel, interferometer, integral hologram, embossed hologram, or computer-generated hologram.

**Fig 3:**
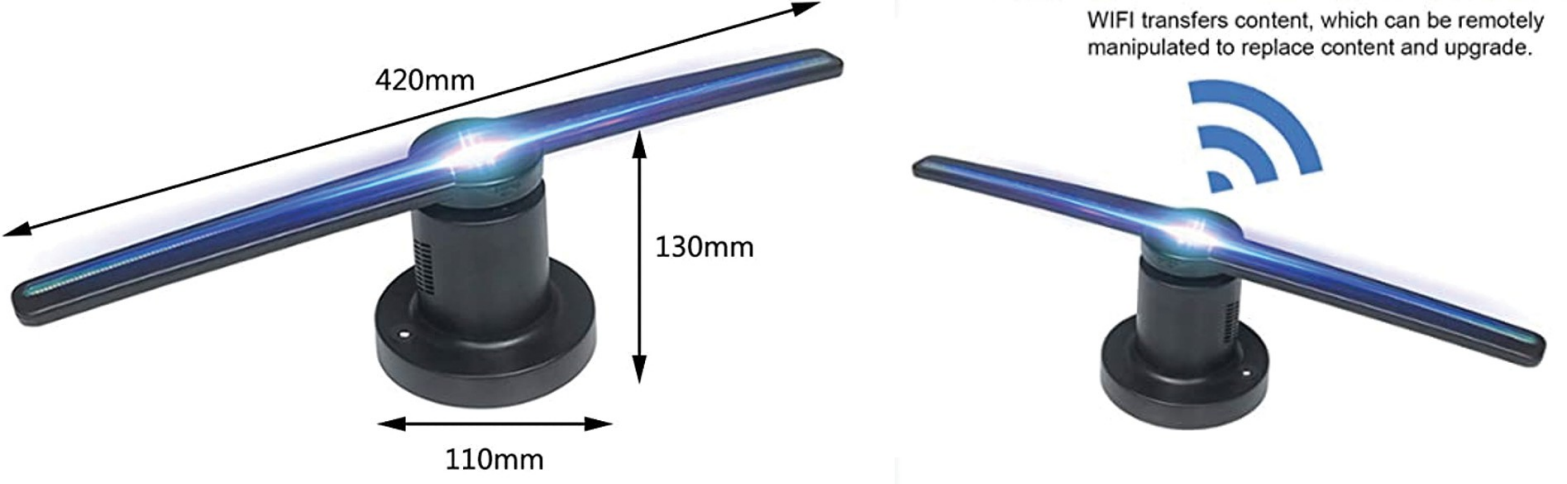


According to Ahmad et al. [50], Augmented Reality (AR) differs from 3DHT in that it displays 3D objects that cannot be seen by the naked eye. It can be experienced by wearing AR glasses or using a smartphone to display a virtual 3D image on the screen. A Hologram LED-fan projector device (HLFP) was used in this study, as shown in (Fig 3). The HLFP was extensively used in the field of education [47, 50, 51]. It is capable of displaying 3D graphics through rapid rotation, but does not produce sound. To improve the impression of 3D graphics on learners, it relies on designing 3D films through computer applications that have a black background. The device is controlled via a mobile phone or computer using Wi-Fi and is easy to use, inexpensive, and high quality.

## Materials and methods

### Ethics statement

The Scientific Research Committee at the Faculty of Physical Education, Port-Said University (Approval No. 2022-7-1), approved the study. Informed consent was obtained from the participants in writing, including voluntary participation, withdrawal rights, objectives, importance, procedures, and confidentiality. At the end of the form, participants can select Agree or Disagree to indicate their agreement to participate in the study.

### Design

**Fig 4:**
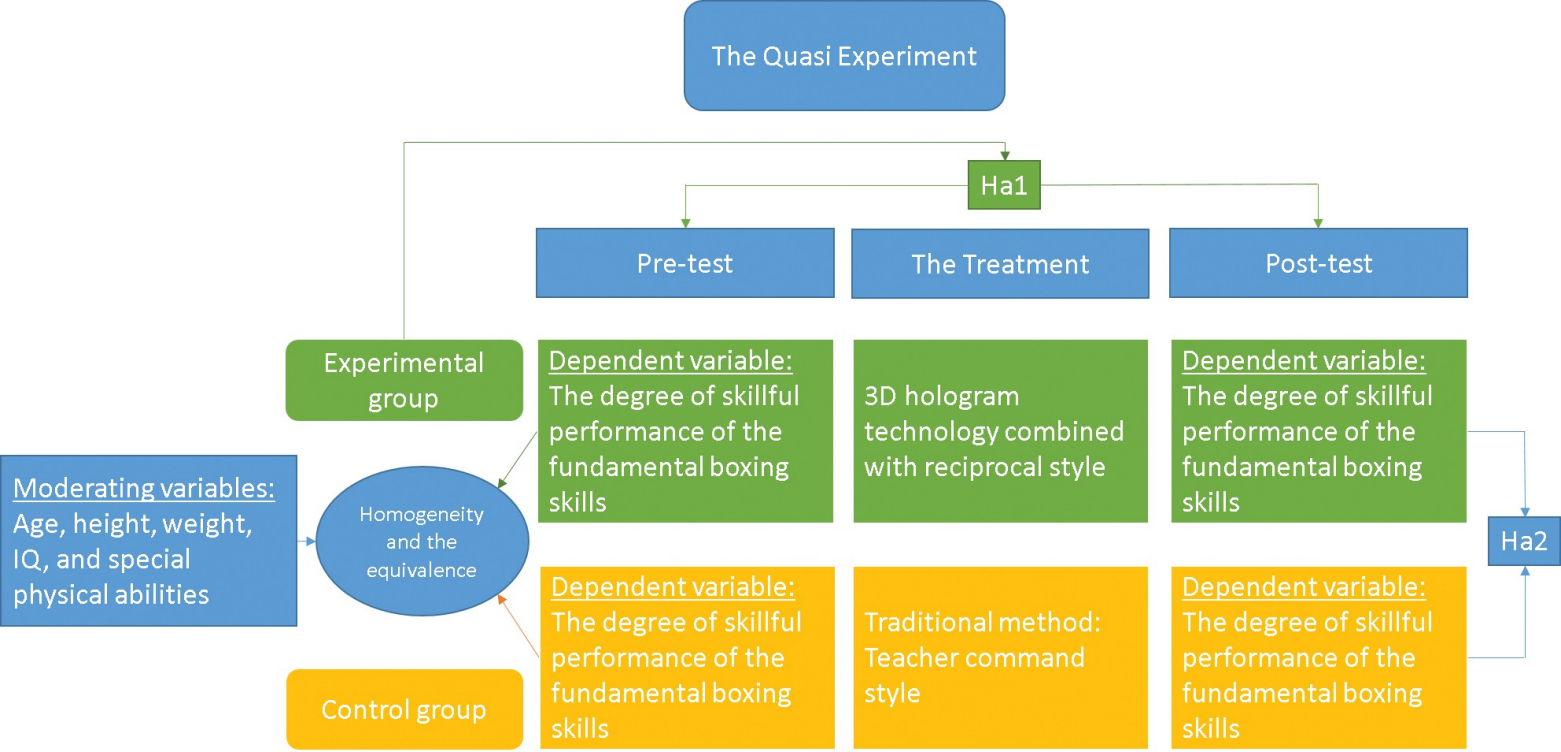


A quasi-experimental design was used by creating two experimental and control groups. For the experimental group, the reciprocal method is combined with 3DHT to teach some basic boxing skills. In contrast, the control group is taught a program based on a teacher command style. Pretest-posttest designs were made for the two groups. The conceptual framework was illustrated in (Fig 4).

### The hypotheses

*H*_*a*1_: In the experimental group, there were statistically significant differences between pre- and post-measurements regarding fundamental boxing skills performance levels in favour of the post-measurements.

*H*_*a*2_: In post-measurements, there were statistically significant differences between the experimental and control groups regarding fundamental boxing skills performance levels in favour of the experimental group.

### Study population and sample

The sample consisted of 40 boxing beginners aged 12 to 14 who were enrolled in the 2022/2023 training season at Port Fouad Sports Club in Port Said, Egypt. The participants were randomly divided into two experimental and control groups. There were homogenized based on age, height, weight, general intelligence, physical fitness, and skill level.

### Data collection tools and equipment

#### The general IQ test

The study used an IQ test developed by Saleh [52]. This is appropriate for the sample age. In this test, children between 8 and 17 are ranked according to percentiles and IQ levels. The test measures general mental ability. There are 60 homogeneous items with five images each. Four images are similar, and one is different. During ten minutes, candidates discover the different images. In studies using the test, reliability coefficients range from 0.75 to 0.85, and validity has been confirmed [53–56].

#### Physical fitness tests for boxers

The present study utilized nine physical tests for boxing sport, which Nasr [57] used on a sample similar to that used in Nasr’s study of boxing juniors aged 12–14. In his analysis, he used reference surveys and expert opinion polls, as well as an exploratory sample. He calculated the reliability coefficient that has been ranged from 0.76 to 0.84, and he validated those tests by applying discriminatory validity. Tests showed strong reliability and validity, indicating they could accurately measure boxing sport physical abilities in juniors aged 12–14. These tests are as follows:

- Kinetic velocity (Throw a heavy sandbag with straight punches left and right in 10 seconds) (Punches in the largest possible number)
- The Power (Right straight punch to throw a 2 kg medicine ball as far as possible) (m)
- The Power (Left straight punch to throw a 2 kg medicine ball as far as possible) (m)
- Reaction Speed (cm)
- Agility (Jumping quadruple in 10 seconds) (Count of jumps)
- Performance endurance (Two minute punching record) (Count of punches)
- Cardio-respiratory Endurance (Running 1500 meters)
- Muscular Endurance (Sit up from lying down) (The largest number possible)
- Muscular Endurance (Push-Ups) (The largest number possible)

**Skill performance evaluation checklists by arbitrators.** Khalifa [58] developed skill performance evaluation checklists which includes the following items::

- Guidelines for technical performance evaluation criteria to determine fundamental boxing skill scores.
- Guidelines for the evaluation phases of fundamental boxing skill.
- Reporting sheet for skills scores.

Many previous studies have used these checklists on similar samples as the current study, including [58–60], which found that they had a validity coefficient of 0.97 and a reliability coefficient of 0.87.

### An educational program combining reciprocal style and 3DHT

A proposed educational program combining reciprocal style with 3DHT was designed using the ADDIE model, in stages as follows:

#### 1. Analysis

At this stage, the program objectives, characteristics of the participants, and educational activities are defined, as follows:

- *The general objective* is to improve the level of proficiency of some fundamental boxing skills for beginners aged 12–14.
- *Characteristics of the participants*: Study participants range in age from 12 to 15 years, which is considered adolescence. This stage is a transition between childhood and adulthood. It begins with secondary sexual characteristics and ends when the adolescent has reached physical and psychological maturity. Among the most significant physical and kinetic changes, the muscular system develops about a year later than the skeletal system. Adolescents become fatigued and exhausted due to it, and rapid growth during the first years of adolescence causes inaccurate movement. Along with the widening of shoulders and enlargement of the buttocks, the torso and legs also lengthen, making the individual stronger and longer. Additionally, the brain also undergoes changes during adolescence, resulting in increased self-awareness, creativity, and social skills [61, 62].
- *Educational activities*: There are two types, one performed by the coach and one by the player:
  - *Coach’s activities*: Coaches explain the objectives of an educational module, prepare players with warm-up and physical preparation exercises, explain the reciprocal learning style, and show a 3D movie of the basic skill using 3DHT. They then follow up on the work of peers, and guiding them towards achieving the desired objectives.
  - *The players’ activities*: The player collaborates and exchanges his roles between a performer and observer, performing tasks listed on work sheets and watching a hologram technique of the skill under study.
- *Educational content*: For the purposes of the study, the following basic skills were chosen, which are considered a starting point for learning the rest of the boxing skills, namely Boxing Stance, footwork, and the four straight punches.

### 2. Design

It includes defining behavioural outcomes, determining the teaching strategy, designing teacher and student guidelines, establishing assessment strategies, and creating collaborative task sheets.

- *Creating behavioural outcomes*: According to Bloom [63], 38 behavioural outcomes were developed: 16 cognitive, 12 psychomotor, and 10 emotional.
- *Teaching Strategies*: The reciprocal style combined with 3DHT was applied with the experimental group. Program modules were designed and each module included learning outcomes, activities, and work sheets for activating the reciprocal style.
- *Designing work sheets*: which provide instructions, outcomes, tasks, performance times, and criteria for evaluating performance. According to Mosston and Ashworth [17], work sheets allow students to actively participate in a task, leading to greater work efficiency and productivity, reducing repetitive explanations, and allowing students to learn how to follow written instructions.
- *Time Framework*: For the experimental group, three educational modules were scheduled per week for five weeks. Module duration is 90 minutes.

### 3. Development

**Fig 5:**
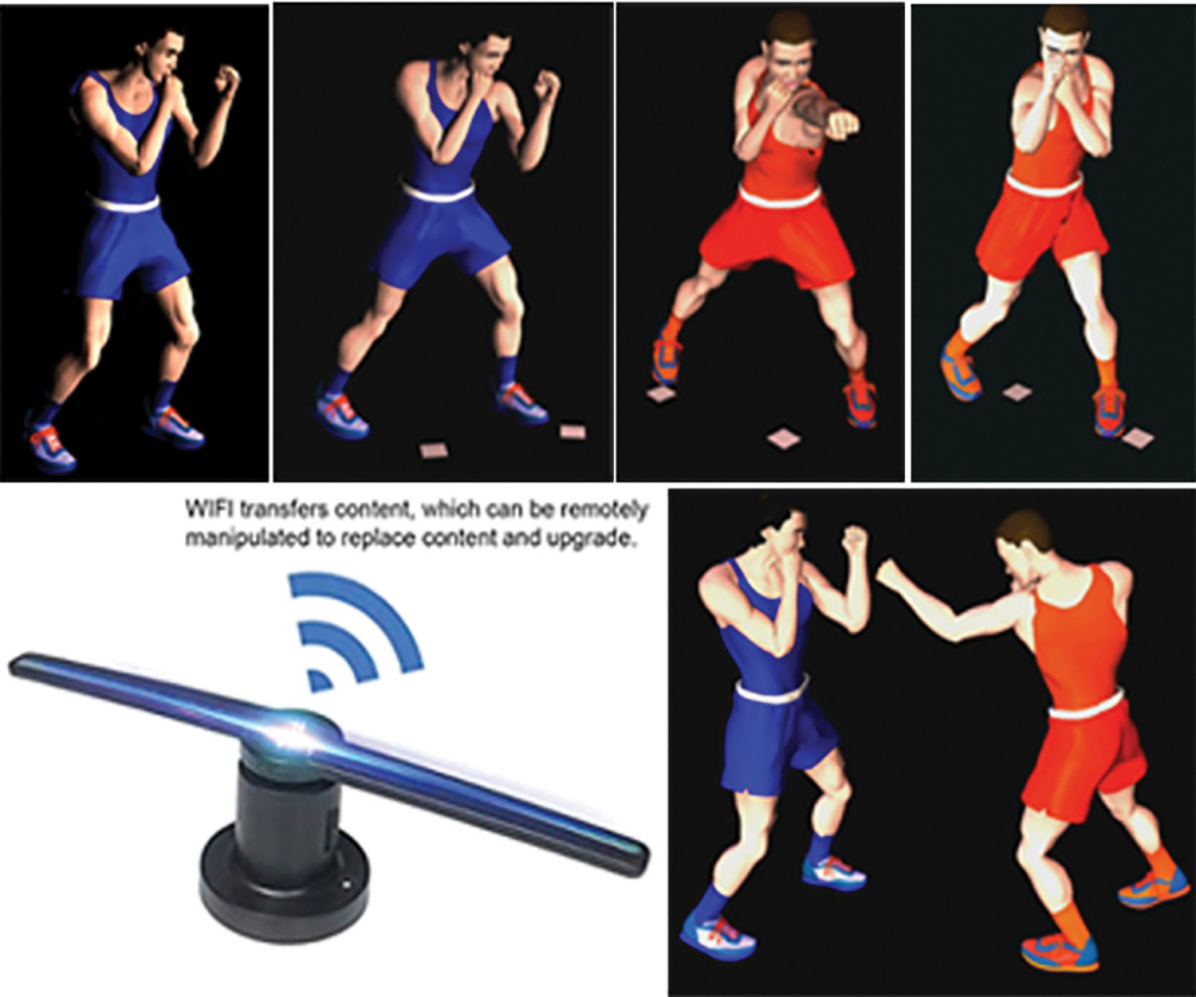


- ***3****D movies design*: In a previous study, the author designed 3D movies using Poser 7 software according to scientific standards for the fundamental skills under study [15]. In the current study, Poser 7 software was used to convert every movie’s background to black to give the effect of holograms on the HLFP device. In this way, distractions that may increase cognitive load on the working memory can be removed, allowing the player to concentrate on technical performance without interference.
- *Uploading and controlling the movies on the hologram fan*: For the current study, three pieces of the Douself 224 Light Beads WiFi 3D Holographic Projector Hologram Advertising Display Fan were provided (Fig 5). The specifications of the device are as follows:

There are 224 light beads.

- Product Dimensions ‏: ‎ 48 x 15 x 12.5 cm
- Resolution(Pixel): 450*224
- Content Format: MP4, AVI, RMVB, MKV, GIF, JPG, PNG
- Support: TF Card and Wi-Fi
- Cool, three-dimensional, holographic, enhance attention, and enhance visual impact.
- WIFI transfers content, which can be remotely manipulated to replace content and upgrade.
- Portable and easy to install.
- Easy to replace content.

**Fig 6:**
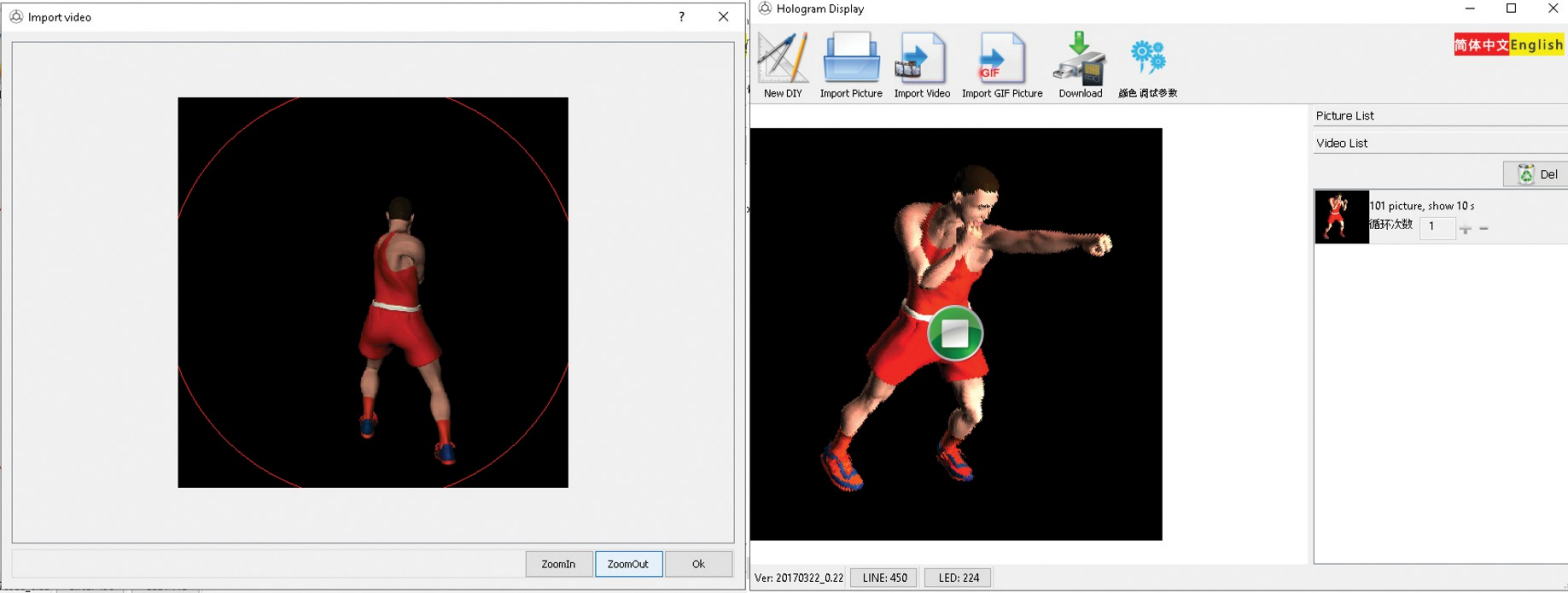


Videos were edited using Holocube X5 software (Fig 6) to ensure that it appeared in the correct size according to the hologram fan’s circumference, and converted from MP4 to Bin format, which the fan recognizes. Moreover, 3D-Player software, which is available in three types (Windows, Android, and iOS), was also used to control the fan via Wi-Fi, upload movies, edit and delete.

**4 Implementation.** The control and experimental groups were given three educational units per week for five weeks, with the experimental group using the reciprocal learning style combined with 3DHT and the control group using the teacher command style.

### The pre-measurements process

At Port Fouad Sports Club, four boxing coaches assisted in taking pre-measurements from the experimental and control groups between 16-17/7/2022, in order to verify the homogeneity and the equivalence of the groups with respect to age, height, weight, IQ, special physical abilities, and the degree of skillful performance of the fundamental boxing skills. The homogeneity of the pre-measurements for each group is depicted in Table 1. Additionally, Table 2 illustrates equivalence between the two groups for those variables.

**Table 1:** Descriptive statistics of the control group and Experimental group (*n*_1_ = *n*_2_ = 20).

Variables	Control group				Experimental group			
	*M*	*SD*	*Skew*	*SE*	*M*	*SD*	*Skew*	*SE*
Height (cm)	150.05	6.62	.17	.51	152.35	8.51	.37	.51
Weight (kg)	57.35	13.81	-.10	.51	55.40	12.72	.08	.51
Age (y)	12.52	.39	.51	.51	12.35	.31	0.59	.51
IQ (Score)	36.7	3.77	-.59	.51	37.75	4.45	.11	.51
Kinetic velocity (Throw a heavy sandbag with straight punches left and right in 10 seconds) (Punches in the largest possible number)	29.35	3.80	.47	.51	30.7	3.80	.41	.51
The Power (Right straight punch to throw a 2 kg medicine ball as far as possible) (m)	4.2	.63	-.13	.51	4.53	.53	.21	.51
The Power (Left straight punch to throw a 2 kg medicine ball as far as possible) (m)	4.13	.62	-.54	.51	4.11	.52	.07	.51
Reaction Speed (cm)	10.65	2.28	.42	.51	11.25	1.65	-.11	.51
Agility (Jumping quadruple in 10 seconds) (Count of jumps)	4.5	1.15	.00	.51	5.05	1.57	-.09	.51
Performance endurance (Two minute punching record) (Count of punches)	126.05	5.45	.06	.51	128.4	4.43	.04	.51
Cardio-respiratory Endurance (Running 1500 meters)	6.71	.88	.58	.51	6.47	.9	.50	.51
Muscular Endurance (Sit up from lying down) (The largest number possible)	31.85	3.98	.95	.51	32.8	4.25	.42	.51
Muscular Endurance (Push-Ups) (The largest number possible)	8.9	2.40	.38	.51	9.5	3	.77	.51
Boxing Stance (Arbitrators’ mean scores)	1.18	.40	-.13	.51	1.17	.38	.02	.51
Footwork (Arbitrators’ mean scores)	1.53	.58	-.02	.51	1.33	.48	0.01	.51
Lead punch to the head (Arbitrators’ mean scores)	2.12	.55	.21	.51	2.17	.38	.02	.51
Lead punch to the Body (Arbitrators’ mean scores)	1.91	.61	-0.16	.51	1.83	.51	-.19	.51
Rear punch to the head (Arbitrators’ mean scores)	1.70	.54	0.32	.51	1.58	.51	-.16	.51
Rear punch to the Body (Arbitrators’ mean scores)	1.51	.41	0.24	.51	1.63	.46	.05	.51

**Table 2:** Independent samples t-test for equality of means between control and experimental groups (*n*_1_ = *n*_2_ = 20).

Variables	Levene’s Test for Equality of Variances		t-test for Equality of Means				
	F	*p*.	*t*	df	*p*	Mean Difference	Std. Error Difference
Height (cm)	2.93	0.10	0.95	38.00	0.35	2.30	2.41
Weight (kg)	0.35	0.56	-0.46	38.00	0.64	-1.95	4.20
Age (y)	0.40	0.53	0.08	38.00	0.93	0.01	0.12
IQ (Score)	1.81	0.19	0.81	38.00	0.43	1.05	1.30
Kinetic velocity (Throw a heavy sandbag with straight punches left and right in 10 seconds) (Punches in the largest possible number)	0.00	0.98	1.12	38.00	0.27	1.35	1.20
The Power (Right straight punch to throw a 2 kg medicine ball as far as possible) (m)	0.00	0.99	1.82	38.00	0.08	0.34	0.18
The Power (Left straight punch to throw a 2 kg medicine ball as far as possible) (m)	0.38	0.54	-0.11	38.00	0.91	-0.02	0.18
Reaction Speed (cm)	2.96	0.09	0.95	38.00	0.35	0.60	0.63
Agility (Jumping quadruple in 10 seconds) (Count of jumps)	3.14	0.08	1.26	38.00	0.21	0.55	0.44
Performance endurance (Two minute punching record) (Count of punches)	0.37	0.55	1.48	38.00	0.15	2.35	1.59
Cardio-respiratory Endurance (Running 1500 meters)	0.10	0.75	-0.85	38.00	0.40	-0.24	0.28
Muscular Endurance (Sit up from lying down) (The largest number possible)	0.47	0.50	0.73	38.00	0.47	0.95	1.30
Muscular Endurance (Push-Ups) (The largest number possible)	0.52	0.48	0.70	38.00	0.49	0.60	0.86
Boxing Stance (Arbitrators’ mean scores)	0.10	0.75	-0.14	38.00	0.89	-0.02	0.12
Footwork (Arbitrators’ mean scores)	1.37	0.25	-1.17	38.00	0.25	-0.20	0.17
Lead punch to the head (Arbitrators’ mean scores)	2.92	0.10	0.34	38.00	0.74	0.05	0.15
Lead punch to the Body (Arbitrators’ mean scores)	1.45	0.24	-0.47	38.00	0.64	-0.08	0.18
Rear punch to the head (Arbitrators’ mean scores)	0.85	0.36	-0.64	38.00	0.52	-0.12	0.18
Rear punch to the Body (Arbitrators’ mean scores)	0.10	0.75	0.85	38.00	0.40	0.12	0.14

Table 1 showed that the skewness values for control group ranged from -0.59 (*SE* = 0.51) to 0.58 (*SE* = 0.51). For experimental group, the skewness values ranged from -0.19 (*SE* = 0.51) to 0.77 (*SE* = 0.51). Therefore, the skewness values for the two groups smaller than the absolute value (1.96*.51 = 0.99), which is acceptable for a normal distribution [64, 65].

The Kolmogorov-Smirnov test was used to verify the normality of the pre-measurements for the control and experimental groups. The test results for the control group ranged from D (20) = .125, *p* = .20 to *D* (20) = .184, *p* = .075, which is greater than the significance level (.05). The results for the experimental group ranged from *D* (20) = .112, *p* = .20 to *D* (20) = .189, *p* = .06, which is also greater than a significance level (.05).). Thus, we accept the null hypothesis that the results were normally distributed for the two groups, and the independent samples t-test can be applied to verify the equivalence between the two groups [65]. Table 2 showed that the F-values for Levene’s test ranged from (0) to (3.14) and the *P*-values ranged from (.09) to (.99) greater than (.05); thus, the variances were not significantly different from one another. Therefore, we can use the *t* value and degrees of freedom. The t-test for independent samples ranged from *t* (38) = -1.17 to *t* (38) = 1.48, and the *P*-values were ≥.05 for all variables. The null hypothesis was accepted, as there were no statistically significant differences between the experimental and control groups in the variables of the pre-measurement, confirming that the two groups are statistically equivalent in those variables.

### The basic experiment process

A boxing coach from the Port-Fouad club delivered three sessions per week to each group starting on 23/07/2022. The teaching period for both groups ended on 24/08/2022. The coach was accredited by the Egyptian Boxing Federation, and has over 10 years’ experience in boxing training. Additionally, he has experience as an international boxer. The author explained to him the reciprocal style and how 3DHT could be integrated into the basic experiment. Furthermore, he explained how the same procedures could be applied to both groups. In order to ensure a proper teaching environment and to monitor progress, the author visited the coach regularly.

### The post-measurements process

Post-measurements were applied immediately after the two groups completed the basic experiment on 25/08/2022. Under the same conditions and timing, arbitrators applied the skill performance evaluation checklists [58] to both groups.

### Statistical analysis

IBM SPSS Statistics for Windows (2017; version 25; IBM Corp, Armonk, NY, USA) was used for the following statistical analysis: Mean (*M*), Standard Deviation (*SD*), Skewness coefficient, Levene’s Test (*F*), Kolmogorov-Smirnov test (*D*), Paired Samples *t*-test, and Independent samples *t*-test.

## Results

The Kolmogorov-Smirnov test was used to verify the normality of the post-measurements for the control and experimental groups as shown in Table 3. Control group results ranged from *D* (20) = .138, *p* = .20 to *D* (20), *p* = .53 which is higher than the significance level (.05). Results for the experimental group ranged from *D* (20) = .152, *p* = .20 to *D* (20) = .190, *p* = .06, which is also above the significance level (.05). Thus, we accept the null hypothesis that the results were normally distributed for the two groups. In this case, an independent samples *t*-test can be applied to verify differences between the two groups in post-measurements, while a Paired Samples *t*-test can be applied to verify differences between the experimental group’s pre- and post-measurements [65].

**Table 3:** A normality test for post-measurements.

Variables	groups	Kolmogorov-Smirnov^a^		
		*D*	*df*	*P*
Boxing Stance (Arbitrators’ mean scores)	Experimental	.162	20	.178
	Control	.185	20	.072
Footwork (Arbitrators’ mean scores)	Experimental	.190	20	.057
	Control	.191	20	.053
Lead punch to the head (Arbitrators’ mean scores)	Experimental	.187	20	.064
	Control	.169	20	.134
Lead punch to the Body (Arbitrators’ mean scores)	Experimental	.171	20	.126
	Control	.157	20	.200
Rear punch to the head (Arbitrators’ mean scores)	Experimental	.184	20	.075
	Control	.123	20	.200
Rear punch to the Body (Arbitrators’ mean scores)	Experimental	.152	20	.200
	Control	.138	20	.200

### Research question 1: Is there a statistically significant difference between the pre- and post-measurements of the experimental group regarding fundamental boxing skills?

*H*_*a*1_: In the experimental group, there were statistically significant differences between pre- and post-measurements regarding fundamental boxing skills performance levels in favour of the post-measurements.

**Fig 7:**
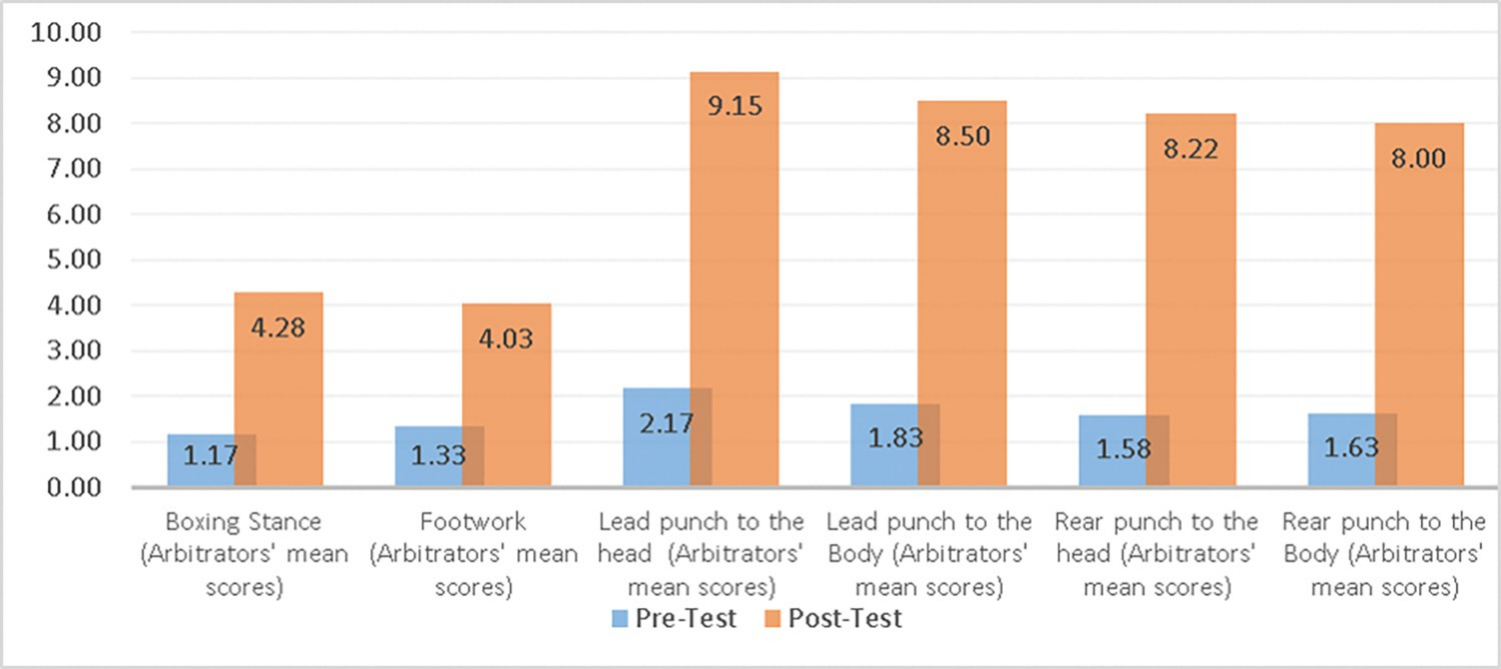


A comparison of pre and post measurements for the experimental group is shown in Table 4 and (Fig 7). The t-values ranged from *t* (19) = 22.37, *p* ≤.05, to *t* (19) = 423.24, *p* ≤.05. Since the *P* value was less than the significance level (0.05), we reject the null hypothesis, so there is a statistically significant difference between pre and post measurements for the experimental group, favoring the post measurements.

**Table 4:** Statistical comparison between pre and posttest for experimental group with paired samples t-test (*n* = 20).

Variables	Test	*M*	*SD*	Paired Samples Statistics		
				*t*	*df*	*P*
Boxing Stance (Arbitrators’ mean scores)	Pre-Test	1.17	0.38	22.37	19	.00*
	Post-Test	4.28	0.44			
Footwork (Arbitrators’ mean scores)	Pre-Test	1.33	0.48	117.57	19	.00*
	Post-Test	4.03	0.52			
Lead punch to the head (Arbitrators’ mean scores)	Pre-Test	2.17	0.38	423.24	19	.00*
	Post-Test	9.15	0.40			
Lead punch to the Body (Arbitrators’ mean scores)	Pre-Test	1.83	0.51	42.48	19	.00*
	Post-Test	8.50	0.38			
Rear punch to the head (Arbitrators’ mean scores)	Pre-Test	1.58	0.61	113.64	19	.00*
	Post-Test	8.22	0.38			
Rear punch to the Body (Arbitrators’ mean scores)	Pre-Test	1.63	0.46	279.06	19	.00*
	Post-Test	8.00	0.48			

*Reject the null hypothesis

### Research question 2: What is the significance of the differences between the experimental and control groups on the post-measurements of fundamental boxing skills? Furthermore, what is the effect size of the reciprocal style combined with 3DHT?

*H*_*a*2_: In post-measurements, there were statistically significant differences between the experimental and control groups regarding fundamental boxing skills performance levels in favour of the experimental group.

**Fig 8:**
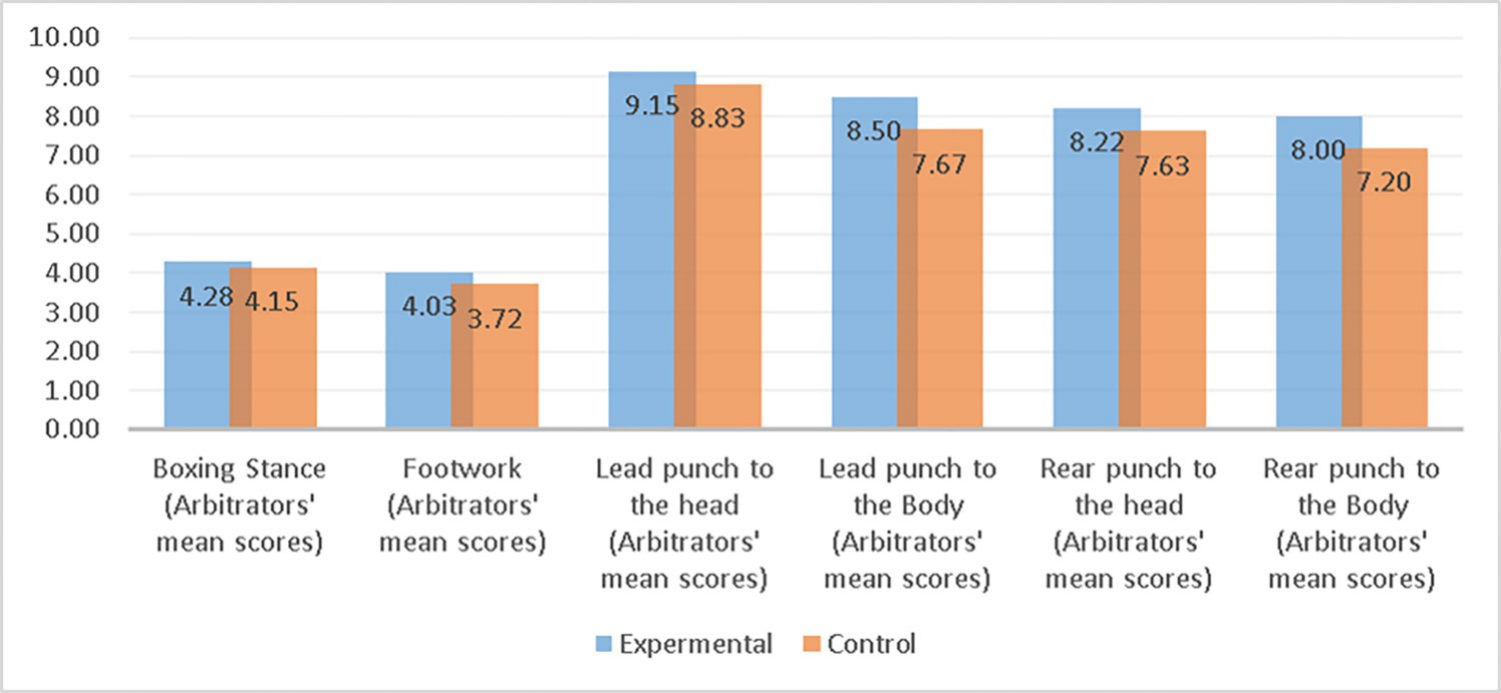


Table 5 showed that the *F*-values for Levene’s test ranged from 0.04 to 4.13 and the *P*-values ranged from 0.05 to 0.85 which ≥ .05. Thus, the variances were not significantly different from one another. In this case, we could use degrees of freedom and the *t* values. For the Boxing Stance skill, the independent samples *t*-test result was *t* (38) = .88, *P* = .38 which > .05. For the Footwork skill, the independent samples t-test result was *t* (38) = 1.98, *P* = .05 which ≥.05. Accordingly, we accepted the null hypothesis that there were no statistically significant differences between the experimental and control groups for those skills at post-measurements. As for the four straight punches, the result of the independent samples *t*-test in the post-measurements ranged from *t* (38) = 2.59, *P* = .01 to *t* (38) = 6.05, *P* = .00, which the *P*-Values < 0.05. Thus, the null hypothesis was rejected, and there were statistically significant difference in the four types of straight punches performance levels between experimental and control groups, favoring the experimental group as shown in Table 5 and (Fig 8).

**Table 5:** Statistical comparison between control and experimental groups`posttest using independent samples t-test (*n*_1_ = *n*_2_ = 20).

Variables	Groups	*M*	*SD*	Levene’s Test for Equality of Variances		Independent samples t-test				
				*F*	*P*	*t*	*df*	*P*	*MD*	Std. Error Difference
Boxing Stance (Arbitrators’ mean scores)	Experimental	4.28	0.44	0.87	0.36	0.88	38.00	0.38	0.13	0.15
	Control	4.15	0.52							
Footwork (Arbitrators’ mean scores)	Experimental	4.03	0.52	0.04	0.85	1.98	38.00	0.05	0.32	0.16
	Control	3.72	0.50							
Lead punch to the head (Arbitrators’ mean scores)	Experimental	9.15	0.40	0.09	0.76	2.59	38.00	0.01*	0.32	0.12
	Control	8.83	0.38							
Lead punch to the Body (Arbitrators’ mean scores)	Experimental	8.50	0.38	0.93	0.34	6.05	38.00	0.00*	0.83	0.14
	Control	7.67	0.48							
Rear punch to the head (Arbitrators’ mean scores)	Experimental	8.22	0.38	4.13	0.05	3.55	38.00	0.00*	0.58	0.16
	Control	7.63	0.63							
Rear punch to the Body (Arbitrators’ mean scores)	Experimental	8.00	0.48	1.35	0.25	4.70	38.00	0.00*	0.80	0.17
	Control	7.20	0.59							

*Reject the null hypothesis

In order to investigate the statistical differences between the two groups, Table 6 revealed that the independent samples *t*-test result was *t* (38) = 3.38, *P* = 0.01 for the overall boxing performance degree. As a result, the null hypothesis was rejected, and there was a statistically significant difference in overall boxing performance between experimental and control groups. Moreover, Table 6 shows that the difference between the two groups’ skill performance degree has an effect size of (*η*^2^ = 0.23). This was greater than 0.14 and denoted a larger effect size. Further, Cohen’s effect size (d) was determined using the following equation [66]:

$$Cohen's\;(d)=\frac{M1-M2}{\sqrt{\frac{(N1-1){SD1}^{2}+(N2-1){SD2}^{2}}{(N1+N2)-2}}}$$

**Table 6:** Statistical comparison between the control and experimental groups’ overall boxing skill degree in the posttest (*n*_1_ = *n*_2_ = 20).

Variables	Groups	*M*	*SD*	Levene’s Test for Equality of Variances		Independent samples t-test					Effect size		Power *1-β*
				*F*	*P*	*t*	*df*	*P*	*MD*	Std. Error Difference	*η* ^2^	*Cohen’s (d)*	
Overall boxing skill degree	Experimental	42.19	2.55	.78	.38	3.38	38	.00*	2.99	.88	0.23	1.07	0.91
	Control	39.20	3.03										

*Reject the null hypothesis

The Cohen (d) value was 1.07, which was greater than 0.8. It confirmed the previous finding of *η*^2^, which also indicated a large effect.

**Fig 9:**
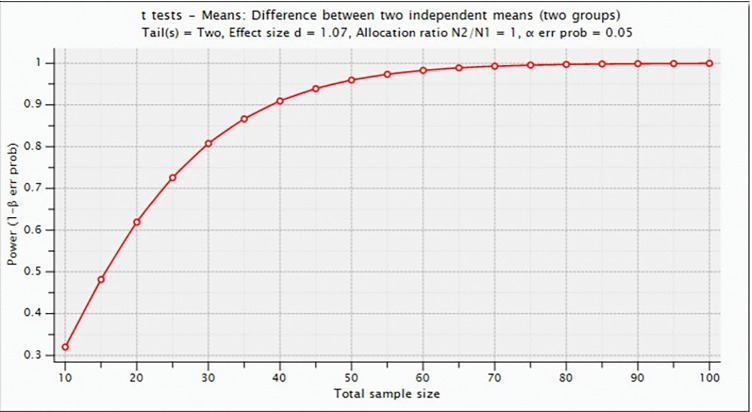


With the use of the G*Power 3.1 tool, a Power Post-hoc analysis was conducted by Cohen’s (d) = 1.07 at a significance level of .05 for independent samples t-test (two groups), while sample sizes for each group were *n*_1_ = *n*_2_ = 20. Consequently, the power (1-*β*) = 0.91 was greater than 0.80, which indicates a true effect size. The effects of the sample can therefore be reliably applied to the whole population, as illustrated in Fig 9 [66–68].

## Discussion

The results of the first question indicated that the experimental group performed fundamental boxing skills better in the post-measurements than in the pre-measurements. Considering that the participants were beginners to boxing, these results demonstrated the effectiveness of the proposed education program that used the reciprocal style by integrating 3DHT. According to the second question, the experimental group performed better in four straight punches than the control group during the post-measurements. These results were due to the reciprocal relationship between the players in the experimental group. This relationship included executing the required tasks using worksheets that allowed the observer player to provide feedback to the performer player. It also included exchanging roles between them, which positively influenced learning straight punches. These results are in agreement with those reported by **Pitsi et al. [19]**, Kolovelonis and Goudas [69], Chatoupis and Vagenas [70], and Kyritsopoulos et al. [71], which indicated that the reciprocal teaching style assisted players in improving their performance because the observer provided feedback repeatedly to the doer. In this way, analyzing the doer’s movements, comparing them with criteria in the worksheet, and reporting conclusions about performance accuracy yielded a greater number of correct responses. The players also developed their social skills. Additionally, these results are consistent with sociocultural constructivist theory, which emphasizes how social situations lead to positive activity in learning **[**25, 26**]**. In the Zone of Proximal Development (ZPD), reciprocal learning and 3DHT integration were the educational scaffolds that assisted the experimental group in understanding how skillful performance is executed and how to perform it perfectly [28]. The educational scaffolding in this study was consistent with what Thomas et al. [72] emphasized in their study, which is that scaffolding represents general guidelines for practice rather than direct recommendations. Furthermore, a scaffolding framework for sports training should be viewed as a progressive social education framework to promote effective learning and development for coaches and athletes. The reciprocal style of learning sports skills is consistent with this.

Moreover, the experimental group’s superiority in post-measurements was attributed to using 3DHT, which created a 3D illusion of the educated skill, attracting students’ attention and allowing them to visualize and monitor how the skill was performed. Additionally, 3DHT enhances spatial capacity, an important factor in education, as the learner should absorb spatial information from sensory memory. This is consistent with previous studies such as Prado Ortega et al. [8], Saito et al. [9], Elmarash et al. [10], and Triberti et al. [11], which agreed with the findings in the current study that 3DHT enhances spatial ability by drawing learners’ attention to 3D objects and enabling them to observe them in the real world without intermediaries. In this way, the virtual object is perceived as a real object moving in a real environment and enhances the learners’ spatial abilities. This provided novice boxers with a model for the ideal motor performance of the learned skill, which was significant in the current study.

The well-designed learning program incorporating 3DHT assisted in placing an appropriate cognitive load in working memory, constructing schemas, and transferring and retaining information in long-term memory, which is consistent with cognitive load theory [30, 31].

Regarding the lack of statistically significant differences between the experimental and control groups in a boxing stance and footwork skills performance degree in the post-measurements, we attribute this to the fact that these skills are first acquired and trained in the first and second educational modules, followed by repetition and correction throughout the entire educational program. This allowed learners to master these skills regardless of the teaching strategy used on them. This is consistent with previous studies such as [58, 59].

The application of 3D hologram technology to teach boxing fundamentals offers an advanced virtual learning environment and provides knowledge that can be applied to real-world scenarios. The visual effect of the 3D hologram captured students’ attention and held their interest, leading to greater motivation among students. The students realized their pre-test mistakes after watching the 3D hologram animation, and in the post-test, the experimental group demonstrated confidence in their ability to perform boxing skills accurately. In this study, participants indicated that they were interested in this kind of learning method compared to conventional teaching methods. Students enjoyed this type of technology and were asked to use it in other courses as well. Several faculty members also expressed excitement about 3D hologram technology when they saw it. Students showed that this technology is beneficial for various reasons, one of which is that it increases their attention span when studying.

Based on the large effect size, the post-hoc power analysis further confirms that the effect size was not due to chance, demonstrating that the difference between the two groups was statistically significant. Therefore, the findings can be generalized to the entire population.

## Conclusions

The purpose of this study was to investigate the effectiveness of 3DHT combined with a reciprocal teaching style for learning some fundamental boxing skills. The experimental group performed fundamental boxing skills better in the post-measurements than in the pre-measurements. The experimental group also achieved higher skill levels than the control group, which relied only on the teacher’s command style. Due to this, it is imperative to utilize hologram technology as a pedagogical resource to enhance the learning process and combine it with teaching strategies that support active learning. Further, the power post-hoc analysis confirmed that the large effect size was not caused by chance, showing that the difference between the two groups was statistically significant. Thus, the findings are generalizable to the entire population.

### Limitations

A WiFi 3D Holographic Projector Hologram is available at electronic stores and is considered one of most accessible and affordable devices. In order to give the illusion that the 3D virtual objects are actually embedded in real life, it uses specialized software to create 3D movies with black backgrounds. The learner should avoid extending their hands too close to the projector in an attempt to grab the 3D virtual objects. Additionally, it does not provide sound, but external speakers can play 3D movies simultaneously through a computer or smart phone.

### Implications

Holograms allow students to interact with 3D virtual objects of motor skills, which helps to improve their understanding of how the body moves and how to perform movements correctly. Additionally, holograms can be used to help players visualize how to combine different elements of a skill and how to link those elements into a smooth and effective performance. This helps to improve player’s spatial awareness and coordination, which makes it easier for them to learn and practice the skill. Therefore, more studies should be conducted to investigate how to integrate hologram technology into education in conjunction with active learning strategies.

## Supporting information

S1 Appendix An example of an educational module used by the experimental group. (PDF)

S2 Appendix The Skill performance evaluation checklists by arbitrators [58]. (PDF)
