# Supplementary material for: Application of 3D hologram technology combined with reciprocal style to learn some fundamental boxing skills
Source: PLoS One. 2023 May 23;18(5):e0286054. doi: 10.1371/journal.pone.0286054 (PMC10204982; doi:10.1371/journal.pone.0286054)
Supplement: S1 Appendix — (PDF) [file pone.0286054.s001.pdf]

Appendix (1): A Sample of the Worksheet used in the First Lecture for the Experimental Group

Instructions for using work sheets

Dear Boxer

To make a good use of the work sheets given to you by your coach during lectures, you need to consider the following:

1- Always use your watch to measure time and a pen to write down finished tasks.
2- Read the paper well before practicing and ask your coach about any unclear things.
3- Be occupied with your work and performance immediately after finishing the training session.
4- Take good care of technical aspect of performing a skill and your coach will correct your mistakes during performance.
5- Use your time to perform the required number of repetitions and use your rest time for finishing unfinished repetitions.
6- Use the 3DHT available in the training hall to know the correct technique of performance.
7- In practices done with a partner, divide work between you and your partner as you make a punch and your partner defenses then change positions.
8- According to the task sheet, the doer boxer performs the task. An observer boxer evaluates the performance of his partner using the criteria sheet, provides feedback to him to correct the mistake, encourages and motivates him to continue working, and then exchanges roles.
9- After finishing work on technique, see your coach to identify your mastery level to move to new skills.

Lecture: One	Week: One	Date: / /	Subject: Technical: stance (25 min) – foot work (forwards – backwards) (20 min)

First: The introductory part of the training module

Subject	Description	Work amount				Notes
		counts	Performance time	Rest time	Total time (min)	
Theoretical lecture and Interaction with 3DHT (20 min)	1- Stance.<br>2- Moving forwards.<br>3- Moving backwards				20	
General warm up (5 min)	Running around the ring and performing general fitness drills to prepare the body				5	
Specific warm up (10 min)	Standing with medical ball beside the body. Jumping over the ball	2	30 sec	30 sec	2	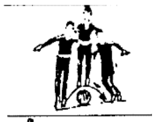
	(Standing) running to cross hurdles by jumping.				2	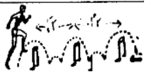
	(standing) hopping in place and rising left foot inwards to touch it with left arm then rotating foot outwards and touch it with the same arm.				2	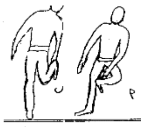
	(Standing – arms beside body) making circles with arms forwards and backwards alternatively.				1	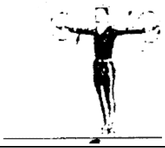

Second: The task sheet for doer boxer

Subject	Description	Work amount				Boxer1		Boxer2		Notes
		counts	Performance time	Rest time	Total time (min)	✓	×	✓	×	
Technique (45 min)	**A) Stance (25 min):**									
	1- taking stance in 4 counts				4					
	2- taking stance in 3 counts				3					
	3- taking stance in 2 counts				3					
	4- taking stance in 1 count				2					
	5- Running in place and taking stance with signal	4	20 sec	10 sec	2					
	6-(Sitting on four) taking stance with signal.	6	10 sec		1					
	7-(laying on back) taking stance with signal	6	10 sec		1					
	8- jumping in place and taking stance with signal	4	20 sec	10 sec	2					
	9- jumping in place and turning backwards then taking stance with signal	4	20 sec	10 sec	2					
	10- jumping in place then making full turn and land to stance with signal	4	20 sec	10 sec	3					
	11- Jumping over a partner`s back and landing to stance.	2	30 sec	15 sec	1.5					
	12- passing through a partner's legs and assuming stance once out	2	30 sec	15 sec	1.5					
	**B) Foot work (20 min)**<br>**1- Moving forwards (10 min)**									
	* Moving forwards in 2 counts. In count (1) move front foot forwards by crawling. In count (2) move rear foot forward with crawl to the same distance				5					
	* Moving forwards in 1 count.				5					
	**2- Moving backwards (10 Min)**<br>* moving backwards in 2 counts. In count (1) move rear foot with crawl backwards. In count (2) move front foot with crawl backwards for the same distance.				5					
	* Moving backwards in one count.				5					
Final part (calming exercises and general feedback) (10 min)										

Third: The Criteria sheet for observer boxer

Skill	Technical criteria for performance	Ideal points	Boxer1	Boxer2
Stance	Keeping head in correct position	1		
	Chine down and look forward	1		
	Correct position of arms and fist	1		
	Trunk right rotation	1		
	Trunk leaning forwards slightly	1		
	Knees bent slightly and body weight distributed on both feet	1		
Moving forwards	Keeping side distance	1		
	Keeping forward and backward distances	1		
	Correct foot moves with crawling	1		
	Speed of foot movement	1		
	Moving on insteps	1		
Moving backwards	Keeping side distance	1		
	Keeping forward and backward distances	1		
	Correct foot moves with crawling	1		
	Speed of foot movement	1		
	Moving on insteps	1		
