# Supplementary material for: Application of 3D hologram technology combined with reciprocal style to learn some fundamental boxing skills
Source: PLoS One. 2023 May 23;18(5):e0286054. doi: 10.1371/journal.pone.0286054 (PMC10204982; doi:10.1371/journal.pone.0286054)
Supplement: S2 Appendix — (PDF) [file pone.0286054.s002.pdf]

Appendix 2. The Skill performance evaluation checklists by arbitrators

First: Technical criteria for evaluating skills

	Technical criteria	Ideal degree of performance
1	Stance	
	- Maintaining the correct head position.	1
	- Maintaining the correct position of arms and fists.	1
	- Rotation of the trunk with a slight forward tilt.	1
	- Bending knees and distributing body weight on feet	1
	- Standing on back foot instep.	1
	Total degree for stance	5
2	Foot work	
	- Keeping forward and backward distances as possible.	1
	- Keeping side distance as possible.	1
	- Moving on insteps.	1
	- Crawling correctly with the foot	1
	- Ideal speed for performance.	1
	- total degree for foot work	5
3	Straight left to head	
	- Extending left arm for the required distance.	2
	- Continuing performance by moving body weight.	1
	- Pelvis and shoulder rotation to the right	1
	- Return of left arm at the same kinetic path for best performance	2
	- Right arm and fist in defense position during performance	1
	- Ideal speed for performance.	2
	- Total shape of performance	1
	- Total degree for straight left to head.	10
4	Straight left to Trunk	
	- Extending left arm for the required distance.	2
	- Bending knees and leaning with trunk to the right	1
	- Pelvis and left shoulder rotation to the right	1
	- Return of left arm at the same kinetic path for best performance	2
	- Right arm and fist in defense position during performance	1
	- Ideal speed for performance.	2
	- Total shape of performance	1
	- Total degree for straight left to Trunk.	10
5	Straight right to head	
	- Extending right arm for the required distance.	2
	- Continuing performance by moving body weight.	1
	- Pelvis and shoulder rotation to the right	1
	- Return of right arm at the same kinetic path for best performance	2
	- Left arm and fist in defense position during performance	1
	- Ideal speed for performance.	2
	- Total shape of performance	1
	- Total degree for straight right to head.	10
6	Straight right to trunk	
	- Extending left arm for the required distance.	2

- Bending knees and leaning with trunk to the left	1
- Pelvis and right shoulder rotation to the left	1
- Return of right arm at the same kinetic path for best performance	2
- Right arm and fist in defense position during performance	1
- Ideal speed for performance.	2
- Total shape of performance	1
- Total degree for straight right to trunk.	10

Second: phases of evaluating skills

Basic skills under investigation.		Phases of evaluating performance.
1	Stance.	Performing skill from still in slow counts with stability in performance
2	Foot work.	Performing skill in slow counts and return to stance.
3	Attack skills.	Performing skill from movement (Shadow boxing).

Third: Evaluation checklists for performing the skills under investigation

Skills		Ideal degree	Student's degree
Basic skills	Stance.	5	
	Foot work.	5	
Attack skills	Straight left to head	10	
	Straight left to torso	10	
	Straight right to head	10	
	Straight right to torso	10	
Total for performance level		50	
